# Primary Hyperparathyroidism in Patients with Multiple Endocrine Neoplasia Type 1

**DOI:** 10.1155/2010/928383

**Published:** 2011-01-17

**Authors:** Grzegorz Piecha, Jerzy Chudek, Andrzej Więcek

**Affiliations:** ^1^Department of Nephrology, Endocrinology and Metabolic Diseases, Medical University of Silesia, ul. Francuska 20/24, 40-027 Katowice, Poland; ^2^Department of Pathophysiology, Medical University of Silesia, ul. Medyków 18, 40-752 Katowice, Poland

## Abstract

Primary hyperparathyroidism may occur as a part of an inherited syndrome in a combination with pancreatic endocrine tumours and/or pituitary adenoma, which is classified as Multiple Endocrine Neoplasia type 1 (MEN-1). This syndrome is caused by a germline mutation in MEN-1 gene encoding a tumour-suppressor protein, menin. Primary hyperparathyroidism is the most frequent clinical presentation of MEN-1, which usually appears in the second decade of life as an asymptomatic hypercalcemia and progresses through the next decades. The most frequent clinical presentation of MEN-1-associated primary hyperparathyroidism is bone demineralisation and recurrent kidney stones rarely followed by chronic kidney disease. The aim of this paper is to present the pathomechanism, screening procedures, diagnosis, and management of primary hyperparathyroidism in the MEN-1 syndrome. It also summarises the recent advances in the pharmacological therapy with a new group of drugs—calcimimetics.

## 1. Introduction

Primary hyperparathyroidism is rarely a part of multiple endocrine neoplasia type 1 (MEN-1) syndrome with familial occurrence. The genetic background of this syndrome offers a unique opportunity to review a pathomechanism of tumourigenesis that may be also operative in some sporadic tumours.

The classic clinical manifestation of MEN-1 is a composition of parathyroid hyperplasia, pancreatic endocrine tumour, and pituitary adenoma [1]. All three tumours, however, do not develop in all affected patients during their life span. Therefore, the contemporary definition of MEN-1 is the coincidence of at least two of the above-mentioned tumours [1]. A diagnosis of familial MEN-1 requires, besides that, a first-degree relative with at least one of the three tumours [1].

In an autopsy series, prevalence of the MEN-1 syndrome was estimated at 2.2 per 1000 in the general population [2], but biochemical surveys suggested lower figures—0.01–0.175 per 1000 [3, 4]. In patients with primary hyperparathyroidism (HPT) approximately 1–5% is associated with the MEN-1 syndrome [3, 5]. Combining this data with HPT incidence, the prevalence of MEN-1 can be estimated to be 10–30 per 100,000 in the general population.

In about 60% of MEN-1 patients enteropancreatic tumours are found. Most of them are small and nonsecreting. The most common hormonally active ones are insulinomas and gastrinomas. Opposite to parathyroid tumours MEN-1-associated gastrinomas are typically multiple, often malignant [6]. Moreover, it is important to stress that other enteropancreatic tumours usually accompany gastrinoma in MEN-1 [7]. Insulinoma is found in 10–30% of MEN-1 cases with the classic clinical presentation with recurrent neuroglycopenia, mainly in fasting, similar to that of sporadic cases. Other rare enteropancreatic tumours diagnosed in MEN-1 may secrete somatostatin, glucagon, vasoactive intestinal peptide (VIP), growth hormone-releasing factor (GHRH), ACTH, or parathyroid hormone-related peptide (PTHrP) [8].

In approximately 30% of MEN-1 patients prolactin-secreting microadenomas or “nonfunctional” adenomas are found in the pituitary gland [9, 10]. Tumours secreting growth hormone or ACTH are less frequent, with the signs and symptoms equivalent to those in sporadic cases.

Neuroendocrine tumour (carcinoid), found in about 14% of MEN-1 cases, originates mainly from the foregut (thymus, bronchus, stomach, pancreas, duodenum) [11], in contrast to sporadic cases originating mainly from midgut [12]. In up to 40% of MEN-1 cases nonfunctional adrenal cortical enlargement has been found by radiological imaging [13]. 

The localization of the tumours in the MEN-1 syndrome cannot be explained by the ubiquitous expression pattern of the mutated gene MEN-1 and its encoded protein, menin. The interaction of menin with mixed-lineage leukaemia protein-containing histone methyl transferase (MLL-HMT) complex mediates tissue-selective tumour-suppressing and tumour-promoting effects and may be responsible for the tissue susceptibility to tumourigenesis in MEN-1 [14].

## 2. MEN-1 Gene Function

The MEN-1 gene is 9.8 kb in 10 exons located on chromosome 11q13 and encodes a 610-amino acid 67-kDa protein menin [15]. Menin is ubiquitously expressed, located primarily in the nucleus [16] and is able to bind to the DNA independently of the sequence [17]. In meiosis it colocalizes with telomeres [18]. Menin is able to bind directly or indirectly to the proteins regulating transcription, DNA processing, or DNA repair as well as cytoskeleton-associated proteins [19–21].

Although its exact role is not fully understood, menin acts as a tumour suppressor. It is suggested that in cells lacking menin DNA damage is increased [22]. Inactivation of the MEN-1 gene causes cell transition from G_0_/G_1_ to S phase and increases their proliferation [23]. Consequently, overexpression of menin induces apoptosis. Loss of this protein prevents apoptosis which normally occurs in cells exposed to UV irradiation or TNF-*α*, and exogenous supplementation of menin restores sensitivity to these stimuli [24]. Moreover, *in vitro *menin overexpression partially suppresses tumour phenotype in neoplastic cell lines supporting its role as a tumour suppressor [25, 26]. Up to now 459 different germline mutations have been reported in the literature and recently summarized [27]. Most of the mutations identified in MEN-1 subjects cause either absence or low availability of menin [28, 29]. A complete loss of menin has been described in tumours from patients with MEN-1 or from mouse models of MEN-1 [30, 31].

A “two-hit” hypothesis is applied to describe the development of tumours in MEN-1 [32]. In a germline carrier of the mutated, nonfunctioning allele a tumour develops after local inactivation of the other allele, allowing clonal initiation and promotion. Sporadic tumours may develop in a similar mechanism as the two alleles are subsequently inactivated in a cell line.

In line with this hypothesis in the sporadic cases of parathyroid adenomas [33, 34], pancreatic [35, 36] and anterior pituitary tumours [37, 38], as well as in carcinoids of lung, thymus and stomach [39, 40], lipomas [38], and skin tumours [41] loss of heterozygosity at the menin locus has been described. Mutation of the MEN-1 gene is the most predominant genetic aberration observed in sporadic endocrine tumours. In approximately 20% of sporadic parathyroid adenoma, gastrinoma, insulinoma, and bronchial carcinoid an MEN-1 mutation can be found [42, 43]. No correlation between MEN-1 genotype and the tumour phenotype or aggressiveness has been found [44], therefore MEN-1 sequencing is not useful for tumour staging.

## 3. Primary Hyperparathyroidism in MEN-1

Primary hyperparathyroidism (PHPT) is the most prevalent clinical expression in MEN-1 mutation carriers, present in more than 90% of cases (Table 1). Most frequently multinodular hyperplasia of parathyroid glands is present; however solitary tumours (usually diagnosed as adenomas) have also been observed. Although the defective MEN-1 gene is a tumour suppressor, the parathyroid carcinoma is diagnosed in the smaller proportion of patients than in sporadic primary hyperparathyroidism. Mild hypercalcemia with normal range serum PTH concentration can usually be detected during the second decade of life [45]. Primary hyperparathyroidism is a progressive disease in MEN-1. Typically the MEN-1-associated parathyroid adenomas are diagnosed at the age of about 25 years [46]—in a considerably younger population compared to the sporadic cases, occurring mainly about the fifth decade of life [47].

A point to be mentioned is the high frequency of supernumerary (up to 20%) and ectopic parathyroid glands, usually localized within the thyroid gland, in the anterior mediastinum and exceptionally in the pericardium in MEN-1 patients [48]. Until now there is no evidence of parathyrematosis in MEN-1-associated PHPT.

The main clinical manifestation of MEN-1-associated PHPT is progressing demineralisation [49] and/or recurrent kidney stones [50, 51]. It was shown that 44% of patients with uncontrolled MEN-1-associated PHPT had severe osteopenia (T score, <−2.0) by 35 years of age [49]. Recurrent kidney stones are less frequently reported in MEN-1 families [50, 51]. Unusually MEN-1 patients develop chronic kidney disease in the course of nephrolithiasis, interstitial nephritis and unrelated to the syndrome diabetes as the most frequent cause of chronic kidney disease all over the world [51, 52].

## 4. Screening for Primary Hyperparathyroidism in MEN-1

It is recommended that biochemical screening for hyperparathyroidism, as well as for the other tumours, should be performed every 1–3 years in the carriers of MEN-1 mutation. For a long period of time clinical manifestation is mild, and the lack of regular screening may result in the delay of diagnosis and numerous complications [50].

Total serum calcium concentration corrected for albumin level or ionized calcium fraction has been considered the single sufficient screening test for hyperparathyroidism in MEN-1 [53], partly because identifying the earliest stages of parathyroid growth has not been considered essential. Recently, however, increased cardiovascular risk has been observed in patients with mild hyperparathyroidism even in the absence of hypercalcemia [54]. This may argue for inclusion of serum PTH concentration measurement as a screening test as well. Diagnosis of hyperparathyroidism requires levels of PTH inadequately high for the parallel calcium levels. At present different kits for assessment of PTH are available. Second generation of kits, measuring concentrations of the so-called “intact PTH” (iPTH), are in the general usage. These tests assess not only the concentration 1-84-PTH, but also of a truncated PTH fragment which was deprived of the first 1–6 N-terminal amino acids that do not stimulate PTH receptor. Until now superiority of the third generation of PTH kits, measuring only the concentration of “native” 1-84-PTH, has not been proven for diagnosis of primary hyperparathyroidism, at least in patients without chronic kidney disease. 

Routine testing for MEN-1 mutation in young patients with primary hyperparathyroidism is not recommended, as these mutations are rare in unselected patients even below 40 years of age [55]. The testing should be considered in patients with additional risk factors such as multiple gland disease, past history, or coexistence of other tumours characteristic for MEN-1, family history of hyperparathyroidism or MEN-1 tumours [56].

## 5. Treatment of MEN-1 Associated Hyperparathyroidism

Total parathyroidectomy and thymectomy with autotransplantation of parathyroid tissue is the therapy of choice for primary hyperparathyroidism in MEN-1 in contrast to single gland-resection in sporadic cases [57, 58]. The surgery in MEN-1-related hyperparathyroidism (HPT) bears also more difficulties: postoperative hypoparathyroidism and higher rates of recurrent or persistent HPT [57]. Recurrence is usually located in preserved parathyroid tissue—either a previously normal gland or a remnant [59]. Subtotal parathyroidectomy or total parathyroidectomy with thymectomy and autotransplantation is associated with fewer recurrences than selective gland excision [60]. The recurrence rate is strongly influenced by proceeding the operation diagnosis of MEN-1, the surgeon's experience, and the possibility to perform an intraoperative histological examination and quick PTH assessment [58]. The main role of intraoperative PTH assessment is to confirm the removal of all the functional parathyroid tissue before parathyroid autotransplantation.

It is not established if the guidelines for the asymptomatic PHPT are applicable for MEN-1-associated PHPT. Some experts advocate early parathyroidectomy to control even mild hyperparathyroidism as uncontrolled disease is associated with a progressive decline of bone mineral density and the increased risk for kidney stones [61, 62]. In addition Burgess emphasises education of the patient in respect to hyperparathyroidism recurrence and possible reoperations in the years to come [61]. 

Because malignancies in MEN-1 parathyroid tumours are uncommon, pharmacological treatment is hypothetically feasible. The development of calcimimetics brought a new effective, however expensive, and until now unlicensed therapeutic option for both sporadic and MEN-1-associated PHPT [63, 64]. Thus, such a therapy seems the only option when surgery is not possible or has been ineffective. Cinacalcet therapy is well tolerated by majority, but not all patients. Gastrointestinal side effects experienced by some patients are followed by discontinuation of this therapy [64]. Additionally calcimimetics have been shown to inhibit parathyroid hyperplasia [65] so one may speculate that they could slow the progression of parathyroid tumours in MEN-1.

## 6. Conclusion

The diagnosis of MEN-1-associated primary hyperparathyroidism should be confirmed by genetic testing. Carriers of MEN-1 mutations require regular screening at least in 3-year intervals regardless of the current presence of hyperparathyroidism from the second decade of life. Surgery remains the main method of primary hyperparathyroidism management despite of high recurrence rate and the necessity of repeated procedures. Calcimimetic agents emerge as new therapeutic option if surgery is not possible or has been ineffective.

## Figures and Tables

**Table 1:** Tumours associated with MEN-1 and their penetrance.

Localization	Clinical manifestation	Penetrance
*Endocrine*		
Parathyroid	Primary hyperparathyroidism (bone demineralisation, kidney stones)	90%
Enteropancreatic		
Gastrinoma	Zollinger-Ellison syndrome (severe peptic ulceration)	40%
Insulinoma	Recurrent neuroglycopenia	10%
Nonfunctioning	Late diagnosis (symptoms related to tumor mass)	20%
Other	WDHA syndrome, diabetes mellitus	2%
Pituitary gland		
Prolactinoma	Galactorrhea, menstrual period anomalies, reduced libido, erectile dysfunction, infertility	20%
Other	Cushing syndrome, pituitary gland insufficiency	17%
Adrenal		
Nonfunctioning cortex	“Incidentaloma”	20%
Pheochromocytoma	Paroxysmal or permanent arterial hypertension, paroxysmal tachyarrhythmia, diabetes mellitus	<1%
Foregut neuroendocrine tumours		
Gastric	Carcinoid syndrome	10%
Thymic	Carcinoid syndrome	2%
Bronchial	Carcinoid syndrome, chronic cough	2%

*Nonendocrine*		
Facial angiofibromas	Local symptoms related to tumor mass	85%
Collagenomas	Local symptoms related to tumor mass	70%
Lipomas	Local symptoms related to tumor mass	30%
Leiomyomas	Local symptoms related to tumor mass	10%
Meningiomas	Intracranial pressure related symptoms	5%
Ependymomas	Local symptoms related to tumor mass	1%
